# A randomized controlled trial of smartphone-based mindfulness training for smoking cessation: a study protocol

**DOI:** 10.1186/s12888-015-0468-z

**Published:** 2015-04-14

**Authors:** Kathleen A Garrison, Prasanta Pal, Rahil Rojiani, Jesse Dallery, Stephanie S O’Malley, Judson A Brewer

**Affiliations:** Department of Psychiatry, Yale School of Medicine, New Haven, CT USA; Departments of Medicine and Psychiatry, University of Massachusetts Medical School, Worcester, MA USA; Department of Psychology, University of Florida, Gainesville, FL USA; 1 Church Street, Room 730, New Haven, CT 06510 USA

**Keywords:** Smoking, Smoking cessation, Mindfulness, Meditation, Smartphone application, App, Mobile health, Craving, Mood, Experience sampling, Ecological momentary assessment

## Abstract

**Background:**

Tobacco use is responsible for the death of about 1 in 10 individuals worldwide. Mindfulness training has shown preliminary efficacy as a behavioral treatment for smoking cessation. Recent advances in mobile health suggest advantages to smartphone-based smoking cessation treatment including smartphone-based mindfulness training. This study evaluates the efficacy of a smartphone app-based mindfulness training program for improving smoking cessation rates at 6-months follow-up.

**Methods/Design:**

A two-group parallel-randomized clinical trial with allocation concealment will be conducted. Group assignment will be concealed from study researchers through to follow-up. The study will be conducted by smartphone and online. Daily smokers who are interested in quitting smoking and own a smartphone (*n* = 140) will be recruited through study advertisements posted online. After completion of a baseline survey, participants will be allocated randomly to the control or intervention group. Participants in both groups will receive a 22-day smartphone-based treatment program for smoking. Participants in the intervention group will receive mobile mindfulness training plus experience sampling. Participants in the control group will receive experience sampling-only. The primary outcome measure will be one-week point prevalence abstinence from smoking (at 6-months follow-up) assessed using carbon monoxide breath monitoring, which will be validated through smartphone-based video chat.

**Discussion:**

This is the first intervention study to evaluate smartphone-based delivery of mindfulness training for smoking cessation. Such an intervention may provide treatment in-hand, in real-world contexts, to help individuals quit smoking.

**Trial registration:**

Clinicaltrials.gov NCT02134509. Registered 7 May 2014.

## Background

Tobacco use is the leading cause of preventable death worldwide [1]. Although over 70% of smokers want to quit, fewer than 5% achieve this annually [2]. Behavioral treatments teach individuals strategies to avoid smoking triggers, or to foster positive affective states, reduce negative mood, reduce stress by exercise or relaxation, divert attention from cravings, substitute other activities for smoking, or to develop social support mechanisms [3,4]. These approaches have only modest success, with 6–12 month abstinence rates of 20-30% [5,6]. Their limited success may be due to the fact that smoking triggers are always present, making avoidance difficult; diversion of attention requires cognitive resources which are often depleted after strong affective states [7]; and effective substitutions are not always readily available.

Recent work suggests that mindfulness training may be an effective behavioral treatment for smoking by targeting stress, craving and learned responses. Mindfulness training has been adapted as a central component of some contemporary psychotherapy and has shown some efficacy in the treatment of psychiatric disorders relating to or involving pain, anxiety, and depression [8,9]. Mindfulness has been operationalized into two components: (i) maintaining attention on immediate experience and (ii) maintaining an attitude of acceptance toward that experience [10]. Mindfulness training typically involves the training of attention regulation, body awareness, and emotion regulation, among other components [11]. In this way, mindfulness training may aid in smoking cessation by teaching individuals to pay attention to and work mindfully with affective states and craving as they arise, rather than to react by smoking.

Preliminary evidence supports testing mindfulness training in the treatment of substance-use disorders and other addictions [9,12]. A recent review found that mindfulness-based interventions were associated with reduced consumption of substances of misuse including alcohol, cocaine, amphetamines, marijuana, cigarettes, and opiates, as compared to several active and inactive control groups [13]. Moreover, several studies included in the review [13] report that mindfulness training was associated with reduced craving and increased mindfulness, suggesting a potential mechanism contributing to outcomes. Although several limitations of prior studies were identified such as small sample sizes and lack of methodological details, findings overall suggest that mindfulness training may be an effective treatment for addictions. A recent randomized controlled trial (RCT) reported preliminary evidence that mindfulness training was associated with increased smoking cessation rates as compared to another leading, widely disseminated and validated smoking cessation treatment [14]. In that trial, home mindfulness practice was also found to reduce the association between craving and smoking [15].

Despite these promising findings, in-person mindfulness training is challenged by the need for experienced therapists, significant demands on time, limited access to and high costs of in-person treatment delivery, and other limitations. One way to overcome these challenges is to offer mobile mindfulness training delivered by smartphone. The global use of smartphones is growing, for example nearly half of U.S. adults (age 18+, 45%) and two-thirds of young adults (age 18–29, 66%) now use smartphones [16] (young adults also have the highest smoking rates in the U.S. (32-35%) [17]). Smartphone app-based treatments for addictions are also gaining popularity due to relative low cost, ease of use, and availability [18,19]. This suggests the demand for and utility of a mindfulness training smartphone app for smoking cessation. In addition to improving treatment dissemination, this approach can be standardized compared to in-person training; may reduce stigma associated with seeking treatment for substance use; improves tracking of adherence such as to home meditation practice, as compared to self-report; offers direct access to tools for self-management and social support; and gets treatment into the hand of the individual in context [18]. Beginning work has evaluated the efficacy of smartphone-based treatments for smoking. A recent pilot trial compared SmartQuit, an app teaching acceptance and commitment therapy, which uses mindfulness strategies, to the National Cancer Institute's QuitGuide app for smoking cessation, and found higher engagement and promising quit rates for SmartQuit [20]. Therefore in this study we translate a mindfulness training program that has shown preliminary efficacy for smoking cessation into a smartphone app.

The smartphone app is also embedded with experience sampling [21] to query individuals about their behavior and experience in real time to measure psychological mechanisms of change *in vivo*. By sampling behavior and experience in real time, it is possible to minimize recall bias, maximize ecological validity, and document change over time [21]. Tracking cigarette use in real time also avoids known biases in retrospective reports [22]. Experience sampling has been used successfully across disciplines including mindfulness [23] and smoking cessation [24], and will be utilized here to evaluate smoking, craving, mood, and mindfulness.

This trial is innovative by: (i) mobile mindfulness training merges a new treatment for smoking –mindfulness training –with a novel delivery method –smartphone app; (ii) mobile mindfulness training can potentially improve efficacy by bringing mindfulness into real-world contexts; (iii) experience sampling will capture real time, real-world smoking, craving, mood, and mindfulness, by smartphone app, whereas prior studies were limited to text messaging or web-based tools [23-26]; and (iv) a novel comparison group, experience sampling-only, will be used to disentangle the efficacy of mindfulness training from that of effective self-monitoring.

The primary aim is to evaluate the efficacy of mobile mindfulness training plus experience sampling to improve smoking cessation rates at 6-months follow-up as compared to experience sampling-only. It is hypothesized that mobile mindfulness training will contribute to a significantly higher smoking cessation rate at 6-months follow-up as compared to control. The secondary aim is to test the hypothesis that mobile mindfulness training moderates the decoupling of craving and smoking. It is hypothesized that mobile mindfulness training will moderate the change in the prediction of smoking by craving across study time points. It is also hypothesized that meditation practice during treatment will moderate this change.

## Methods/Design

A pragmatic RCT funded by the American Heart Association will be conducted. Participants will be allocated to either the control (experience sampling-only) or intervention (mobile mindfulness training plus experience sampling) group by basic randomization, using Yale Qualtrics Survey Tool (http://www.qualtrics.com/). Ethical approval for the procedures of this trial was obtained from the Yale University Institutional Review Board Human Investigation Committee (HIC #1301011337).

### Setting

The study will be conducted online and by smartphone. Treatment delivery will be smartphone-based. Participants (*n* = 140) will be recruited using online advertising (e.g., http://www.google.com/ads/) and directed to the study website. This recruitment method has been used to register 89 participants and randomize 16 participants per week [27]. This approach will have broad reach.

### Participants

Participants will be eligible if they are: age 18–65 years; smoke at least 5 cigarettes per day; less than 3 months abstinence in the past year; own an iPhone (https://www.apple.com/iphone/) or Android (https://www.android.com/intl/en_us/); and are motivated to quit, as indicated by a score of at least 9 of 10 on the Contemplation Ladder [28], and at least 4 of 5 on one item of the Action subscale of the Readiness to Change Questionnaire [29]. “I am trying to smoke less than I used to” from 1=Strongly disagree to 5=Strongly agree” ”. If smartphone ownership is found to be a barrier to recruitment, smartphones and/or voice/data plans will be provided.

### Recruitment and randomization

All study procedures will be automated using Yale Qualtrics Survey Tool. The study website links to a short screening survey. The survey notifies an individual if they are eligible or not eligible. If eligible, they are asked for their contact information and directed to an online consent form, which includes study contact information to ask questions. Those who provide consent are sent an email with a link to the baseline survey and a copy of the consent form. This email also includes a recommendation for nicotine replacement therapy or other pharmacotherapies [19]. At the end of the baseline survey, participants are randomized to treatment group and emailed a link to download their app and begin the study.

### Retention strategies

Treatment retention will be defined as completing 75% of mobile mindfulness training modules. Retention rates will be ensured by: (i) emphasizing the importance of follow-up at study initiation and through follow-up; (ii) requesting three referrals at study initiation who can update a participants’ contact information; (iii) paying $10 for the end of treatment survey, $20 each for surveys at 3- and 6-months follow-up, and $0.50 per experience sampling "check-in" [24]; and (iv) paying for participation (up to $116 total) at 6-months follow-up. All randomized participants will be followed up and their true smoking status used in the analysis, and follow-up will be collected blind to group allocation [30].

### Sample size

The sample size was determined based on the prior study of in-person mindfulness training for smoking cessation [14]. Cell size was determined by a *χ*^*2*^ power analysis using one-week point prevalence abstinence at end of treatment and at 4-months follow-up from that study. Point prevalence abstinence differed between groups by 20% or more at each time point. Thus it is estimated that group sizes of 59 per group will achieve 80% power to detect a 20% group difference at *p* < .05 using a two-sided *Z*-test with pooled variance. 140 subjects will be recruited to allow for 12-15% attrition. Further, a sample size of 33 was sufficient to detect a significant prediction of smoking by craving at baseline and significant effects of meditation on smoking at end of treatment [15], therefore these group sizes will be sufficient to detect similar changes.

### Intervention and comparator

#### Intervention group

The intervention group will receive a smartphone app, Craving to Quit (www.cravingtoquit.com) which delivers mindfulness training and experience sampling daily for 22 days. An introductory email will explain that the app will help participants self-monitor their smoking habits, recognize when and how often they smoke, identify triggers for smoking, and learn methods to become more mindful of cravings and ‘ride them out’. Upon downloading the app, participants will input the number of cigarettes smoked per day and set a quit date of 3 weeks from today. They will view a tutorial explaining the app features and begin the first day.

Mobile mindfulness training is comprised of 22 modules, 5–15 minutes each, designed to teach mindfulness for smoking cessation using audio, video, animations, and *in vivo* exercises (Table 1). Participants have access to only one new module per day; subsequent days are locked to prevent skipping ahead. Four bonus modules become available across the study to bolster training. Overall the concept of mindfulness –paying attention to and accepting momentary experience –will be taught. Participants will learn three standard meditation practices: body scan, loving kindness, and breath awareness [31]. Body scan is practiced by bringing awareness to different parts of the body, and is considered to foster awareness of body sensations that constitute cravings and affective states. Loving kindness is practiced by directed well-wishing by repeating phrases such as "may X be happy,” and is considered to foster acceptance of oneself and others. Breath awareness is practiced by paying attention to the breath wherever one feels it most strongly in the body, and is considered to help retrain the mind away from habitually engaging in self-related thinking toward a more present-centered awareness.

**Table 1:** **Mobile mindfulness training for smoking cessation modules and bonus module contents**

**Module**	**Contents**
1	Introduces the Craving to Quit program and mindfulness, introduces habit formation using an animation, and provides a guided mindful smoking exercise.
2	Setting personalized goals, e.g., list three reasons why you want to quit smoking, and provides a mindful smoking exercise.
3	Teaches body scan meditation, provides a guided body scan exercise, and provides a mindful smoking exercise.
4	Teaches how to work with cues, affective states, and craving using RAIN, and provides a RAIN exercise.
5	Introduces the concept of craving using an animation with the metaphor of craving as a tantrum toddler, i.e., let the toddler cry it out, and provides a RAIN exercise.
6	Teaches how to recognize triggers, e.g., list some of your triggers, and provides a RAIN exercise.
7	Expands on the concept of craving using an animation with the metaphor of craving as a fire, i.e., let the fire burn out, and provides a RAIN exercise.
8	Teaches how to use noting practice, i.e., the “N” of RAIN, in everyday life, and provides a noting practice exercise.
9	Teaches strategies for staying on track, and provides a noting exercise.
10	(Un)resistance Training, and provides a noting exercise.
11	Builds on noting practice by teaching curiosity, a core element of mindfulness, and provides a curiosity exercise.
12	Expands on the concept of craving and curiosity using an animation with the metaphor of a hot coal, asks “What do you get from smoking mindfully today?”
13	Teaches loving kindness meditation, provides a loving kindness exercise, and provides “Wild Geese” by Mary Oliver.
14	Teaches evaluating the costs & benefits of smoking, provides a loving kindness exercise.
15	Misperceptions about Quitting, tell a smoking buddy you are quitting today
16	Builds on noting and curiosity by teaching noting while walking meditation, provides a walking noting practice.
17	Teaches open awareness of thoughts, to work mindfully with thoughts that trigger smoking, using animations such as “Thoughts like a Radio.”
18	Builds on walking while noting with animations such as “Tripping on Thoughts,” “Autobiography in 5 short chapters” by Portia Nelson, provides a noting exercise.
19	Asks subjects to reflect on their own experience with treatment to gather evidence for how treatment helps with smoking cessation, noting practice with a particular eye out for doubt.
20	Provides tips on staying motivated and maintaining mindfulness practice, subjects write down a mantra to use and set mantra reminder.
21	Quit day ceremony, subjects tell a friend or family member that today is their quit day.
22	Incorporates mindfulness practices as a new, healthy habit, and instructs the user on which modules to return to if they relapse.
Bonus	“Big Mind Meditation” audio by Joseph Goldstein; Tree Analogy for reinforcing noting video; Attitude is Everything video; “Mountain Meditation” audio by Joseph Goldstein; Sitting Meditation audio

Participants will also be taught an informal mindfulness practice to work mindfully with cravings: Recognize, Accept, Investigate, and Note (“RAIN”) what cravings feel like as they arise and pass away. RAIN is accessible from the home page of the app so that participants may use it any time they have a craving to smoke by choosing the “Want-O-Meter.” They are directed to identify their smoking trigger, rate their craving, and choose between using RAIN to ride out their craving, or completing an audio-guided exercise to ‘smoke mindfully’ by paying attention to the moment-to-moment experience and bodily sensations of smoking.

Social support has been shown to boost quit rates [32], therefore the app is also paired with a closed online community, built on the Ning platform (www.ning.com). Features include: (1) video tutorials of how to use the app; (2) quit buddy sign ups, for individuals to go through the process with a peer; (3) forums for posting questions and insights; (4) a “Tip of the week;” and (5) an “Ask the Doc” feature to get answers from an “expert” (JB). Participants can access the community beyond their quit date in order to encourage others to quit and to bolster their own abstinence.

Additional app features include: (i) “Activity Feed” tracks interaction with the app and can be shared on the community; (ii) “My Morning Stats” tracks daily smoking to encourage a gradual taper, and daily and monthly financial savings; (iii) “Night Reflection” to reflect on daily smoking, with links to journal on the community and practice an audio-guided body scan or loving kindness meditation; (iv) “Reminders Settings” to adjust times/days to receive reminders including “Morning Stats Motivator,” “Goal Reminder,” “Check-ins,” and “Night Reflection;” (v) “My Quit Pact” which can be personalized across the intervention and includes quit date, money saved, reasons for quitting, triggers to watch for, and a personalized mantra; and (vi) “Tracker,” to track cigarettes smoked.

Experience sampling is embedded in the app as “check-ins.” Based on previous experience sampling methods for smoking cessation [33], participants will be prompted six times per day to check-in, each day of the 22-day program. They will set the daily start and end times for check-ins, their day will be divided into six intervals, and they will be prompted to check-in at a random time in each interval [23]. Each check-in will ask the following questions adapted from the Day Reconstruction Method [34] and from prior studies using experience sampling [23,24]: (1) When you started this check-in, what were you doing? Categorical response (traveling, working, eating, internet/texting, relaxing, reading, talking, grooming, housework, watching TV, shopping/errands, caring for children, preparing food/eating, exercising, other). (2) When you started this check-in, how aware were you of what you were doing? Visual analog scale (VAS) from not at all to very much. (3) When you started this check-in, how distracted or focused were you? VAS from very distracted to very focused. (4) When you started this check-in, how was your experience feeling? VAS from very unpleasant to very pleasant. (5) When you started this check-in, how were you relating to your experience? VAS from really wanted it to stop to really wanted it to continue. (6) When you started this check-in, how much were you craving a cigarette? VAS from not at all to very much. (7) When you started this check-in, how much were you feeling these emotions? VAS from not at all to extremely, for each: relaxed, anxious, joyful, tired, content, sad, excited, irritable. (8) The tracker says you have smoked (#) cigarettes today. Adjust your tracker below if needed. Today I have smoked: (#). Participants will be sent a reminder text message if their response rate drops below 3 check-ins in 24 hours. Any day with less than 3 completed check-ins will be excluded from the analysis. Based on prior studies [23] a response rate of 80% is expected.

Upon completion of the 22-day program, participants will receive an email of congratulations, reminding them that they will be paid for the check-ins they completed across the program 6-months from when they started the study, and that they will receive the next survey 1-month from when they started the study (the dates will be provided as a reminder). Links to the online follow-up surveys will be emailed to participants at 1-, 3-, and 6-months. At 6-months follow-up, participants who report one-week abstinence from smoking will take part in carbon monoxide (CO) monitoring. CO levels will be measured using piCO+ Smokerlyzer breath CO-monitors (www.bedfont.com). CO-monitors will be shipped to participants with usage instructions, and they will set up a recorded, smartphone-based video call with a researcher, during which they will use the CO-monitor and show the output to the researcher [35]. They will be provided a paid shipping return label and will be asked to return the CO-monitor in its original packaging. Once a participant has completed the 6-months follow-up survey and returned the CO-monitor (if applicable), they will be emailed an Amazon (www.amazon.com) gift card with their study payment.

#### Control group

The control group will receive experience sampling-only, to control for: (i) expectancy effects of treatment for smoking; (ii) non-specific effects of using a smartphone for smoking cessation; and (iii) potential effects of experience sampling for smoking cessation. Experience sampling may bring awareness to craving and smoking, and is similar to other interventions that teach individuals to identify triggers and track the number of cigarettes they smoke. Experience sampling-only will be delivered by smartphone app for 22 days. An introductory email will explain that the app will help individuals self-monitor their smoking habits, recognize when and how often the smoke, and identify triggers for smoking. Upon downloading the app, participants will input the number of cigarettes smoked per day and set a quit date of 3 weeks from today. They will view a tutorial explaining the app features and begin the first day. Across treatment, experience sampling procedures will be matched to the intervention group. From day 22, all study procedures will be matched to the intervention group. In addition, along with study payment at 6-months, this group will receive a free download code for the treatment app.

### Measurements

#### Primary outcome measure

The primary outcome is one-week point-prevalence abstinence from tobacco smoking at 6-months, verified by carbon monoxide monitoring [36,37] with the interviewers blinded to treatment allocation. Abstinence rates will be compared between groups using *χ*^*2*^ tests. This trial will use an intention-to-treat approach [30].

#### Secondary outcomes

The secondary outcomes are smoking, craving, mood and mindfulness as measured by experience sampling. Smoking will be measured using the Tracker. Craving will be measured using the check-in question "When you started this check-in, how much were you craving a cigarette?" Prediction of smoking by craving will be tested using standard regression analyses with group and craving as independent variables and smoking as the dependent variable [15]. Formal (meditation) and informal (RAIN) practice will be included in the model as independent predictor variables [15] to test whether meditation practice during treatment moderates the change in the prediction of smoking by craving across study time points.

#### Exploratory outcomes

Theory-based potential mechanisms will be tested using mediation analyses of variables related to smoking, craving, mood and mindfulness, and other psychological variables, evaluated using 15-minute online surveys at baseline, 1-, 3-, and 6-months from treatment initiation, including: (i) smoking demographics, including self report of smoking abstinence over the follow-up period allowing up to five cigarettes total [30]; (ii) 4-item Alcohol Use Disorders Identification Test (AUDIT-C; [38]); (iii) 15-item Minnesota Nicotine Withdrawal Scale-revised (MNWS-R; [39]) to evaluate nicotine withdrawal symptoms (i.e., craving, irritability, anxiety, depression, difficulty concentrating, appetite, insomnia, restlessness, impatience, constipation, dizziness, coughing, dreaming/nightmares, nausea, sore throat); (iv) 4-item Perceived Stress Scale (PSS; [40]) to measure the degree to which situations in one's life are appraised as stressful over the last week; (v) 12-item Smoking Self Efficacy Questionnaire (SEQ; [41]) to evaluate the self-efficacy to resist the urge to smoke; (vi) 24-item Five Facet Mindfulness Questionnaire short form (FFMQ-SF; [42,43]) to measure mindfulness related to observing, describing, acting with awareness, non-judging of inner experience, and non-reactivity to inner experience; (vii) 12-item Self Compassion Scale short form (SCS-SF; [44,45]) to evaluate how one typically acts toward oneself in difficult times; (viii) 22-item Craving Experience Questionnaire (CEQ; [46]) to measure desire and craving to smoke; (ix) exercise, eating, and relaxation methods questions, including prior meditation experiences; and (x) 6-item Gratitude Questionnaire (GQ-6;500 [47]) to evaluate one's proneness to experience gratitude in everyday life. Participants will be paid $10 for the 1-month survey, and $20 for each 3- and 6-months survey, the total to be paid at 6-months.

## Discussion

This trial is the first to test smartphone app-based mindfulness training to for smoking cessation. Although many meditation-related smartphone apps are available, very few have been tested for use in clinical groups such as smokers [18]. In addition, there are currently few smoking cessation interventions that target the association between craving and smoking [12]. In-person mindfulness training has been found to improve smoking cessation rates and decrease the association between craving and smoking [14]. Therefore, this trial will test whether mobile mindfulness training is associated with similar outcomes.

The choice of a control group for this trial was critical for isolating and testing variables of interest, by comparing mobile mindfulness training to experience sampling-only. Alternative comparators could be in-person mindfulness training, an online smoking cessation program, waitlist control, or another smartphone-based smoking cessation intervention. However, there is limited evidence for the efficacy of existing smartphone apps for smoking cessation, and other apps offer additional features not available in the tested intervention that introduce confounds, or vice versa. Experience sampling may bring awareness to smoking, craving, and mood, similar to mindfulness training, therefore experience sampling-only was chosen as the control, to disentangle the effects of mindfulness training from those of effective self-monitoring. Exploratory analyses will test whether experience sampling-only also leads to mindfulness, and whether increases in mindfulness are associated with smoking cessation.

If the intervention is found to be effective, this RCT will provide a platform for large-scale clinical trials to compare mobile mindfulness training to active behavioral treatments for smoking cessation, and to disseminate this treatment to the wider community.
